# Resolutions Respecting a Medical Reform

**Published:** 1809-10

**Authors:** Thomas Taylor


The subject of a Reform in Medicine being again introduced, we are desired to lay the following Papers before our Readers.

*Horncastle, Bull Inn, July* 27, 1809.

AT the Aniversary of the Lincolnshire Benevolent Medical Society, held this day, Dr. Fawssett, the president, in the chair; when the business of the society was concluded, Dr. Harrison addressed the meeting on the indispensable necessity of an improved regulation in the education and admission of future practitioners; especially for the provincial districts of the United Kingdom, which being, under the present laws, out of the jurisdiction of the Royal Colleges of Physicians and Surgeons, are therefore most in want of medical enactments. He also stated what had already been done to attain this important object; and that the bill, now on the table, for regulating medical education tion and practice, was intended, if a sufficient fund could be raised in the mean time, to be presented to parliament in the approaching session.

Mr. Pettener, of Louth, the president for the ensuing year, then read the bill to the members present; after which, the following resolutions were moved by Dr. Bousfield of Spalding, and unanimously adopted.

*First,* That the bill, as now corrected, appears to be well calculated to serve the best interests of the community, by supplying all parts of the empire with confidential practitioners, as the present establishment retire from their functions; and also, by restoring the faculty to the full enjoyment of their profession; in preventing future candidates from entering upon medical practice, until they have been properly educated and examined.

*Second,* That the bill, by suppressing the most dangerous empirics, and imposing restraints upon the rest, will be productive of immediate advantages to society at large.

*Third,* That it appears to this meeting, that the amount of the present subscriptions is quite insufficient for carrying this bill through parliament, and the meeting thinking it desirable to accomplish the same by voluntary contributions, are of opinion, that if all who are impressed with the necessity of the measure, will undertake to exert themselves in their respective neighbourhoods, an adequate fund may be easily raised—since, after providing for the general defence of the country, the preservation and recovery of health should claim the first consideration, from all ranks and descriptions of people.

*Fourth,* That the thanks of this meeting be given to Dr. Harrison for the address he has just read, which it recommends him to prepare for publication; also for his unwearied endeavours to obtain an improved condition of the profession, and which the meeting hopes he will not remit, until the object is fully accomplished.

*Fifth,* That a copy of these resolutions be delivered to Dr. Harrison for the committee of medical practitioners in London, who have co-operated with him in this business; authorizing them to make such use of the same as they may think proper.

THOMAS TAYLOR.

